# Editorial of Special Issue “Multiple Sclerosis: From Diagnostic Biomarkers to Imaging and Clinical Predictors”

**DOI:** 10.3390/diagnostics12020482

**Published:** 2022-02-13

**Authors:** Dejan Jakimovski, Robert Zivadinov

**Affiliations:** 1 Buffalo Neuroimaging Analysis Center (BNAC), Department of Neurology, Jacobs School of Medicine and Biomedical Sciences, University at Buffalo, State University of New York, Buffalo, NY 14203, USA; rzivadinov@bnac.net; 2 Center for Biomedical Imaging at Clinical Translational Science Institute, University at Buffalo, State University of New York, Buffalo, NY 14203, USA

Multiple sclerosis (MS) is a chronic, neuroinflammatory and neurodegenerative disease of the central nervous system (CNS) that can present with a plethora of physical and cognitive impairments [1]. This Special Issue of *Diagnostics* informs the reader on the implementation of multiple conventional and non-conventional diagnostic procedures that can range from the use of clinical predictors and various neuropsychological test batteries to the more advanced use of magnetic resonance imaging (MRI) sequences and positron emission tomography (PET). Given that the measurement of cognitive reserves and determining the extent of cognitive impairment in MS patients is emerging as one of the most prudent research problems, multiple articles in this Special Issue focus on this topic.

The manuscript by Jerković et al. validated a slightly adapted cognitive processing speed test called the letter digit substitution test (LDST) [2]. When compared to the current gold standard of the Symbol Digit Modalities Test (SDMT), LDST replaces the symbols with randomized letters of the alphabet and measures the processing speed over 60 s. An example of the LSDT can be directly accessed through the Supplementary Material of their manuscript [2]. Using LSDT data acquired in 196 healthy controls and 87 MS patents, the Authors determined that 35 points (AUC 0.698 and 95% CI of 0.677–0.718) would represent the optimal diagnostic cut-off point that could differentiate both groups [2]. The LDST demonstrated concurrent and divergent validity when compared to the previously validated MS impact scale (MSIS-29) and the routinely utilized Expanded Disability Status Scale (EDSS). Future studies should determine the impact on the measurement derived from the slight differences between SDMT (use of symbols) and LDST (use of letters). While LDST only requires participants to learn the letter–number combinations, the SDMT requires familiarization to previously unknown abstract symbols.

In addition to the traditional cognitive domains, such as processing speed, executive function and memory, recent MS studies have also focused on exploring the potential deficits in social cognition. The manuscript by Ziccardi et al. is one of the first MS works to provide a longitudinal perspective on this topic [3]. In general, social cognition refers to the person’s ability to infer the emotions, intentions and beliefs of others through recognizing facial emotions and employing empathy. A group of 26 RRMS patients with no evidence of baseline cognitive impairment were followed over 3 years and demonstrated relatively stable social cognition scores. Albeit no impairment in social cognition was noted, and the social cognition scores were significantly associated with both the volume of the amygdala and the volume of cortical lesions [3]. The cortical lesion volume was the only significant predictor of social cognition performance measured 3 year later. The lower social cognitive performance was also correlated with poorer daily well-being measures, such as depression, anxiety, fatigue and quality of life, associated with social functioning [3]. 

As a segue into the imaging block of the issue, two additional manuscripts utilized MRI-based data in order to determine which MS patients are at greater risk of cognitive impairment and future cognitive decline. The first work by Motyl et al. analyzed 1091 MS patients from the Grant Quantitative (CQ) study, which had neuropsychological (measured with SDMT) and MRI examinations at baseline and 12 months afterwards [4]. Interestingly, up to 44 neurologically stable MS patients experienced significant worsening in SDMT (isolated cognitive decline), and more than half (59.1%) of them had concurrent radiological disease activity (new or enlarging T2 lesions) [4]. The presence of higher EDSS scores and lower whole brain volume at the baseline visit were both predictors of isolated cognitive decline. Moreover, 40% of these patients were already classified as cognitively impaired at baseline [4]. The findings from this work are two-fold. The first one is related to the clinical use of cognitive screening with tests such as the SDMT to potentially detect radiologically active MS patients that would otherwise not exhibit any other neurological worsening. Secondly, early detection of cognitive impairment can identify patients that are at risk for additional future cognitive decline.

On a similar note, the manuscript by Bernabéu-Sanz et al. employed voxel-based morphometry (VBM) and diffusion tensor analysis of MRI scans and demonstrated a significant association between lower SDMT performance and lower gray matter volumes in the angular and supramarginal gyri [5]. These findings corroborate the function of these specific cortical areas that are tasked with the function of integrating somatosensory, visual and auditory inputs and utilizing them towards higher cognitive processes. Additional cortical areas for motor coordination, language processing and executive function were related to the SDMT performance [5]. In general, all aforementioned cortical areas are converging and underlying the importance of the frontoparietal attention network in the cognitive processing performance of MS patients. Worse fractional anisotropy values of all thalamic connections and lower streamline values of the inferior fronto-occipital fasciculus were associated with poorer SDMT performance. Lastly, streamlines of the uncinate fasciculus, inferior longitudinal fasciculus and corpus callosum were associated with verbal episodic and semantic memory performance [5]. 

The manuscript by Poulsen et al. aimed at determining the distribution of MS lesions throughout the length of the spinal cord and how their detection can influence the diagnosis of MS [6]. Out of the 74 newly diagnosed MS patients, 58 already fulfilled the dissemination in space (DIS) criteria with just brain MRI scans alone [6]. When the cervical spinal cord scans were added, an additional 9 patients satisfied the DIS criteria. On the other hand, lesion analysis in the thoracic and lumbal spinal cord did not result in a significant increase in DIS diagnosis. The addition of cervical spinal cord imaging did not significantly influence the fulfillment of the dissemination in time (DIT) criteria when compared to the brain-MRI-only protocol [6]. Based on their findings, cervical cord scanning does improve the DIS evaluation and should be implemented in the process of MS diagnosis. On the other hand, additional scanning of the remaining spinal cord provides much lower sensitivity.

Previous studies have shown that MS patients exhibit abnormal CSF flow dynamics when compared to HCs [7]. In this Special Issue, Laganà et al. explore the effect of different analysis software packages on the aqueduct of Sylvius (AoS) segmentation and the CSF flow measures [8]. Based on findings of 30 MS patients and 19 HCs, different image analysis software provided significantly discrepant AoS cross-sectional areas and mean diastolic peak velocities [8]. On the other hand, the maximal diastolic peak flow was a good group differentiator, and these differences were not dependent on which analysis software was used [8]. The Authors conclude that future clinical studies should first determine the repeatability of the analyses before interpreting the study outcomes.

The wide range of symptoms that can occur in MS patients are showcased through two manuscripts by outlining the rate of kinesiophobia and neurogenic lower urinary tract dysfunction. Wasiuk-Zowada et al. administered the Tampa scale of kinesiophobia questionnaire (quantifying the fear of movement) to 81 MS patients, and the kinesiophobia diagnosis (>37 points on the questionnaire) was present in 18.5% of patients in the low-disability category to 74.2% and 65.2% in the more-disabled patients [9]. Based on these findings, MS rehabilitation studies should account for the high kinesiophobia rate in more-disabled MS patients, which could significantly influence the recruitment rate and persistence in the program. Beck et al. performed undynamic studies in 207 MS patients and showcased that the majority (83%) of patients had pathological outcomes indicative of neurogenic lower urinary tract dysfunction [10]. The urodynamic pathology was not dependent on the physical disability of the MS patients [10]. The presence of post-void residual, less than 250 mL voided volume and presence of urinary tract infection in the past 6 months were all associated with neurogenic lower urinary tract dysfunction [10].

In order to treat the neuroinflammatory pathology in MS, multiple anti-inflammatory disease-modifying therapies (DMTs) have been developed with various levels of efficacy and safety properties [11]. The manuscript by Subramaian et al. explores the effect of natalizumab (antibody against α4-integrin that blocks the leukocyte adhesion and their migration through the blood–brain barrier) on CSF-based and MRI-based inflammatory markers [12]. In the 93 MS patients that had data before and after natalizumab initiation, natalizumab resulted with a significant decrease in CSF leukocytes, protein level, albumin quotients and CSF-based oligoclonal bands [12]. In nine patients, the oligoclonal bands disappeared completely after natalizumab treatment [12]. The strong natalizumab-induced changes in CSF inflammatory metrics were concurrently occurring with the reduction in relapse activity and stabilizing the disability worsening, T2 lesion volume accrual and the rate of brain atrophy [12].

Lastly, the work by Jewells et al. utilizes novel in vivo detection of microglia through PET detection of 18-kDa translocator protein (TSPO) radiotracer in demyelinating mice model of MS [13]. TSPO, also called the peripheral benzodiazepine receptor (PBR), is highly expressed in activated microglia and a known prognostic factor in MS pathology. For this purpose, the Authors develop a specific 18F-PBR-111 radiotracer and demonstrated significant uptake after demyelination that was spatially correlated with positive histopathological staining of CD11b (activated microglia) and F4/80 (macrophages) [13]. This increase in uptake was greater when compared to control mice and located in the corpus callosum and striatum [13]. Therefore, PET 18F-PBR-111 could have potential as an in vivo imaging biomarker for MS neuroinflammation and an assessment of microglial activity.
